# Prevalence, laterality, and comorbidity of hippocampal sclerosis in an autopsy sample

**DOI:** 10.1002/brb3.66

**Published:** 2012-06-26

**Authors:** Chris Zarow, Michael W Weiner, William G Ellis, Helena Chang Chui

**Affiliations:** 1 Department of Neurology, USC/RLANRC7601 E. Imperial Hwy. Medical Science Bldg, 90242, Downey, California; 2 Department of Medicine, Radiology, Psychiatry, and Neurology, University of CaliforniaSan Francisco, California; 3 Department of Pathology, University of CaliforniaSan Francisco, California; 4 Department of Neurology, University of Southern CaliforniaLos Angeles, California

**Keywords:** Hippocampal volume, MRI, neurology, neuroscience, TDP-43

## Abstract

Hippocampal sclerosis (HS) is a common and often asymmetric neuropathological finding among elderly persons who experience progressive memory loss, but its cause is unknown and it is rarely diagnosed during life. In order to improve both understanding and diagnosis of late-life HS, bilateral hippocampi and cerebral hemispheres were reviewed in 130 consecutive autopsy cases drawn from a longitudinal study of subjects with subcortical ischemic vascular dementia (IVD), Alzheimer disease (AD) and normal aging. HS was found in 31 of 130 cases (24.5%). Of these, 45% were bilateral, 32% left-sided, and 23% right-sided. The majority of HS cases involved the entire rostral-caudal extent of the hippocampus. However, in 7 cases HS was focal in nature and was only found at or anterior to the lateral geniculate nucleus. In 77% of cases, HS was accompanied by other types of pathology (‘mixed’ HS), but in 23% of cases it was the sole neuropathologic finding (‘pure’ HS). TDP-43-positive cytoplasmic inclusions were found in dentate granule cells in 93% of all HS cases, 55% of AD cases with no HS, but 0% of IVD cases with no HS. MRI hippocampal volumes were significantly lower in bilateral HS compared to AD (*p* < 0.001) and in unilateral HS cases compared to cases with intact hippocampi (*p* < 0.001). Since HS may occur unilaterally in approximately a quarter of cases, its prevalence may be underestimated if only one cerebral hemisphere is examined. The presence of TDP-43 inclusions in HS cases, regardless of accompanying pathologies (e.g., AD, IVD, FTLD), is consistent with an underlying neurodegenerative pathogenetic mechanism. Further studies are warranted to determine whether greater severity of hippocampal atrophy on MRI may assist the clinical differentiation of HS from AD.

## Introduction

Hippocampal sclerosis (HS) describes a condition of severe neuron loss with gliosis, in the absence of cystic cavitation, usually in the CA1 sector and subiculum of the hippocampus. It is commonly recognized as medial temporal sclerosis in young individuals with temporal lobe epilepsy. In elderly persons, HS is an often unrecognized cause of cognitive decline, typically presenting with severe memory loss, but usually not discovered until autopsy. Hippocampal atrophy, characteristic of both HS and Alzheimer disease (AD), is observed on premortem MRI (Jagust et al. 2008b). HS can occur unilaterally or bilaterally and can be complete, extending through the entire rostral–caudal extent of the hippocampus, or focal. Histologically, there is often a sharp demarcation between HS and the adjacent normal hippocampal subfields. In late-life HS, the CA4, CA3, and CA2 subfields of the hippocampus are typically spared. Although HS can be the sole pathological finding (Jellinger 2000), HS can also be associated with many other pathological conditions including AD, vascular dementia (VaD), and fronto-temporal lobar degeneration (FTLD; Zarow et al. 2008). In FTLD-U-associated HS, ubiquitin-positive cytoplasmic TDP-43 immunoreactive inclusions have been described in dentate granule cells (Cairns et al. 2007). Asymmetric and focal involvement of the left and right hippocampi by HS has been recognized, but has rarely been systematically studied. Often only a limited numbers of sections of the hippocampus taken from one hemisphere are available for pathologic evaluation. Only two case-series studies have made explicit mention of the laterality of HS. Leverenz et al. (2002) described a community-based study (*n* = 134) comparing the clinical and pathologic characteristics of HS to AD. Probst et al. (2007) reviewed a series of 10 cases of HS dementia and found three bilateral, five left-only, and two right-only HS. In this study, we systematically describe the prevalence, laterality, and comorbidity of HS in an autopsy series drawn from a prospective, longitudinal MRI study of subcortical ischemic vascular dementia (IVD) and AD.

## Methods

### Sample selection

Cases were obtained from the IVD Program Project, a prospective, longitudinal study of subjects with subcortical ischemic vascular disease (SIVD), AD, and cognitively normal elderly subjects. The sample consists of 139 consecutive autopsies obtained over the 11-year span of 1997 to 2007. The 130 cases with bilateral hippocampi available for review comprise the sample in this study. In most cases (*n* = 121, = 93%), the entire extent of the hippocampus from pes to tail was evaluated by systematic sampling from four to seven consecutive 5-mm-thick blocks spanning the entire hippocampus. In nine cases, only two levels of the hippocampus were reviewed for one or both hemispheres: at the level of the pes and the level of the lateral geniculate nucleus.

**Figure 1:**
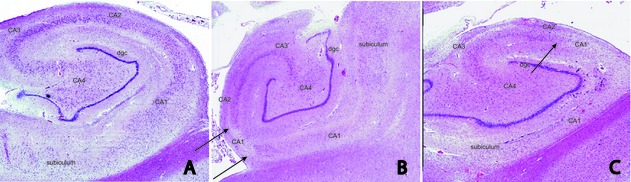
(A) Low-power image of an H&E-stained section of an intact hippocampus with subfields indicated from a 91-year-old female. There is a full complement of neurons in all subfields. (B) Low-power image of an H&E-stained section from a 93-year-old female with focal HS at the CA2–CA1 transition, extending only partially into CA1 (arrows). (C) Low-power image of an H&E-stained section from a 91-year-old female illustrating complete HS. There is an abrupt and dramatic loss of neurons beginning at the CA2–CA1 transition (arrow) and extending throughout the CA1 and the subiculum. dgc, dentate granule cell layer.

HS was evaluated with the H&E stain (Fig. 1), where this lesion is characterized by severe loss of pyramidal neurons and accompanying gliosis. Severity of HS was scored as none, focal, or complete based on the extent of hippocampal involvement. HS was rated ‘none’ when there was no HS, ‘focal’ when HS was limited to a portion of a CA sector at a single level of the hippocampus, and ‘complete’ when HS involved the entire pyramidal layer of CA1 and/or subiculum throughout the rostral–caudal extent of the hippocampus.

### Standardized neuropathologic evaluation

Cases were evaluated for neurofibrillary tangle load (Braak and Braak score), neuritic plaque burden (CERAD rating), vascular brain injury including macroscopic-, lacunar-, and micro-infarcts (cerebrovascular parenchymal pathology scores (CVD-PS); Chui et al. 2006), and Lewy bodies (McKeith Lewy body score; McKeith et al. 1996). Blood vessels were rated for severity of cerebral amyloid angiopathy (Vonsattel rating [grade 0 to III, expanded to grade IV where there is evidence of CAA-associated microangiopathy]; Vinters and Vonsattel 2001). In this study, cases were categorized as AD if the Braak and Braak score was ≥V. IVD was operationally defined as CVD-PS score ≥20; diffuse Lewy body disease (DLBD) as Lewy Body score ≥3. A diagnosis of FTLD was based on a consensus between two board-certified neuropathologists using criteria which included a pattern of atrophy consistent with FTLD, confirmed by appropriate microscopic findings.

### Immunohistochemistry

TDP-43 immunostaining was carried out bilaterally on a subset of cases (*n* = 45) representing normal hippocampus (seven cases), IVD without HS (six cases), AD without HS (18 cases), and HS with and without comorbidities (14 cases). Ten-micron-thick sections of formalin-fixed, paraffin embedded tissue from hippocampus were immunostained for TDP-43 (Proteintech Group, Chicago, IL, 1:50 dilution). The slides were treated with 10 mmol/L sodium citrate pH 4.5 for 10 min at 98°C, prior to overnight incubation with the primary antibody at 4°C. Immunohistochemistry was performed using the Vectastain ABC system (Vector Laboratories, Burlingame, CA) with 3,3-diaminobenzidine as chromogen. Slides were counterstained with hematoxylin for 10 s. Five to seven sections, spanning the entire rostral–caudal extent of the hippocampus from pes to tail, were evaluated for each hippocampus. Cases were judged to be positive if dentate granule cell neurons contained perinuclear cytoplasmic TDP-43 immunoreactive inclusions. The number of TDP-43-positive dentate granule cells was counted for each section. The frequency of TDP-43 cytoplasmic inclusions in dentate granule cells were rated 0 to 3+ on a logarithmic scale as follows: 0 = 0; 1–10 = 1; 11–100 = 2+; >100 = 3+.

Quantitative measures of hippocampal volumes (HV) from 1.5 Tesla premortem MRI were available for 101 of 130 cases, as part of the parent longitudinal study (Jagust et al. 2008a). HV were obtained using a semiautomated high dimensional brain-warping algorithm (Medtronic Surgical Navigation Technologies, Louisville, CO; Du et al. 2006). When more than one MRI was available for analysis, the MRI closest to death was selected. The mean interval between MRI and death was 3.0 ± 2.1 years.

## Results

This cohort of 130 cases with bilateral hippocampus available for postmortem review included 65 cases with pathological diagnoses of AD (51 ‘pure’ AD, 12 mixed AD/IVD, and two mixed AD/DLBD), 28 cases with IVD, seven with DLBD (including 1 mixed DLBD/IVD), 19 pathologically normal, two cases with FTLD, one each multiple sclerosis, progressive supranuclear palsy, pure cerebral amyloid angiopathy, and seven ‘pure’ HS. Among the 18 cases without significant pathologic findings, there were eight subjects who were cognitively normal and 10 who had mild cognitive impairment at the last clinic visit.

**Figure 2:**
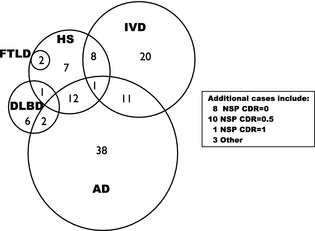
Venn diagram depicting the distribution of HS cases within each pathologic group for 130 autopsy cases. The circle diameters reflect the relative size of each cohort. NSP, no significant pathology; CDR, clinical dementia rating scale.

We found a total of 31 (23.8%) cases with HS, including seven ‘pure’ HS and 24 ‘mixed’ HS (Table 1; Fig. 2). Compared to 18 cases with no significant pathologic change and 81 non-HS cases with other neuropathologic diagnoses, the HS cases were older (analysis of variance [ANOVA], *P* < 0.05) and had fewer years of education (ANOVA, *P* < 0.05; Table 2). Compared to 81 non-HS cases with other types of brain pathology, HS cases had lower brain weight (*t*-test, *P* < 0.05), but there were no statistically significant differences in the proportion of females, the average age of symptom onset, or the duration of illness (Table 2).

**Table 1:** Demographics, laterality, and comorbidities of 31 HS cases

Case	Gender	Age	Education (years)	Sections reviewed	HS	TDP-43 score	HS - right	TDP-43 counts R	MRI hip volume R	HS - left	TDP-43 counts L	MRI hip volume L	CDR	ApoE	Braak & Braak score	CERAD score	MCKEITH score	Brain weight (g)	Final neuropathologic diagnosis
1	M	89	7	10	R-focal	1+	Focal	0	1391.18	None	4	1312.86	3	34	V	Moderate	0	1050	AD+HS
2	F	85	12	11	L-focal	2+	None	0	1475.95	Focal	40	1407.10	4	33	VI	Frequent	0	1050	AD+HS
3	F	86	12	10	B-complete	1+	Complete	68	836.25	Complete	7	935.44	1	na	V	Sparse	0	1070	AD+HS
4	M	86	16	6	B-complete	na	Complete	na	1131.08	Complete	na	1266.65	3	34	VI	Frequent	0	1100	AD+HS
5	M	79	20	4	B-complete	na	Complete	na	1335.92	Complete	na	1334.81	1	24	V	Frequent	0	1240	AD+HS
6	M	87	12	11	R-complete	0	Complete	0	1680.31	None	0	2319.06	0	33	V	None	0	1413	AD+HS
7	F	90	6	7	B-complete	2+	Complete	37	834.83	Complete	45	633.94	3	34	VI	Frequent	0	890	AD+HS
8	F	82	14	8	L-complete	na	None	na	1690.72	Complete	na	1318.72	1	33	IV	Moderate	0	1100	AD+HS
9	F	93	16	6	L-complete	na	None	na	1462.07	Complete	na	1126.05	2	na	VI	Moderate	0	1000	AD+HS
10	F	92	10	8	R-focal	na	Focal	na	2248.79	None	na	2352.36	0	34	VI	Sparse	0	1260	AD+HS
11	M	88	16	7	L-complete	na	None	na	1510.19	Complete	na	994.24	2	34	VI	Frequent	1	1150	AD+HS
12	F	97	16	8	R-focal, L-complete	2+	Focal	93	1047.00	Complete	81	1358.63	2	na	VI	Frequent	0	1000	AD, IVD, CAA
13	M	95	10	7	B-complete	na	Complete	na	766.48	Complete	na	1157.73	3	33	VI	Frequent	0	1170	AD, IVD, CAA
14	M	82	16	12	R-complete, L-focal	2+	Complete	55	1168.82	Focal	52	1294.15	3	34	IV	Sparse	0	1250	HS+CAA
15	M	78	16	7	L-complete	na	None	na	828.79	Complete	na	729.82	1	33	0	None	7	1120	HS+DLBD+IVD
16	M	63	12	10	L-focal	na	None	na	2660.11	Focal	na	1823.24	3	33	0	None	0	1150	HS+FTLD
17	M	71	14	4	R-complete	na	Complete	na	1134.94	None	na	2046.43	0	33	I-II	None	0	1290	HS+IVD
18	M	87	10	12	R-complete	2+	Complete	80	1225.50	None	0	1750.02	2	33	0-I	None	0	1220	HS+IVD
19	F	85	17	10	L-focal	na	None	na	2076.29	Focal	na	1021.47	0	33	III	Mild	0	990	HS+IVD
20	F	88	13	8	B-complete	na	Complete	na	1143.00	Complete	na	969.00	2	33	II	Moderate	0	1050	HS+IVD
21	M	75	12	8	R-focal	na	Focal	na	2113.56	None	na	1939.00	1	22	I	None	0	1180	HS+IVD
22	M	89	17	9	R-focal, L-complete	2+	Focal	12	1303.32	Complete	48	1270.84	1	na	IV	Moderate	0	1300	HS+IVD+CAA
23	M	88	12	8	L-complete	na	None	na	1064.25	Complete	na	815.69	2	24	I	Sparse	0	1220	HS+IVD+CAA
24	M	90	16	8	L-complete	na	None	na	1520.91	Complete	na	877.04	2	33	III	Sparse	0	1210	HS+IVD+CAA
25	M	86	12	7	B-complete	3+	Complete	124	1504.48	Complete	365	1085.93	2	na	III	Sparse	0	1350	Pure HS
26	F	77	13	10	B-complete	na	Complete	na	911.96	Complete	na	1163.75	2	34	III	Sparse	0	1135	Pure HS
27	M	78	8	11	B-complete	3+	Complete	553	902.32	Complete	673	1489.66	3	33	0	None	0	865	Pure HS
28	F	88	17	10	B-complete	2+	Complete	58	na	Complete	10	na	1	na	IV	Sparse	0	1360	Pure HS
29	M	87	16	7	R-focal, L-complete	3+	Focal	168	712.75	Complete	44	641.06	2	33	III	Sparse	0	1142	Pure HS
30	F	92	13	7	R-complete	na	Complete	na	1868.22	None	na	2131.10	2	33	I	Sparse	0	1250	Pure HS
31	F	93	12	11	L-focal	2+	None	4	1333.25	Focal	15	1334.69	0.5	33	III	Sparse	0	1075	Pure HS

**Table 2:** Group characteristics for 130 consecutive autopsy cases

		Non-HS		
			
	HS	Non significant pathology	Other pathologic diagnoses	ANOVA (*P*)
*N*	31	18	81	
% Female	41.9	72.2	38.3	
Age (SD)	82.9 (7.29)	83 (6.1)	81.5 (7.3)	0.04
Age range	63–97	74–91	63–95	
Age of onset	74.2 (8.15)	82 (3.8)	73.6 (8.3)	0.013
Duration	7.43 (4.5)	3.6 (1.8)	7.7 (4.4)	0.051
Education	13.0 (3.2)	14 (4.0)	15.2 (3.3)	0.034
Brain weight (g)	1150 (131)	1238.9 (131.2)	1211.1 (175.8)	0.11
CDR	1.76 (1.07)	0.28 (0.3)	1.67 (1.05)	0.004

Age onset for NSP comes from a few cases which had CDR = 0.5, but did not have significant pathological findings anywhere in the brain.

### Pathological comorbidities with HS

There were seven (22.6%) cases in which HS was the only significant pathological finding (so-called ‘pure’ HS). More commonly (77.4%), HS was found in association with other neuropathologic diagnoses (Table 1; Fig. 2). Of 31 HS cases, 12 also met neuropathologic criteria for AD, eight had IVD, one had mixed AD/IVD, one had mixed DLBD/IVD, and two had FTLD. HS was found in roughly equal proportions in AD and SIVD (24% and 29%, respectively).

### Laterality of HS

Bilateral HS was present in 14 (45.2%) of 31 cases, with variable combinations of severity on each side (Table 1). HS was present only in the right hippocampus in eight (22.6%) cases (four complete and three focal), and present only in the left hippocampus in 10 (32.2%) cases (seven complete and three focal).

### Extent of HS

As noted in the methods section, cases were designated ‘complete’ when HS involved the entire pyramidal layer of CA1 and/or subiculum throughout the rostral–caudal extent of the hippocampus. Ten cases had bilateral complete HS. Four additional cases were bilateral, but were complete on one side and focal on the other. Ten cases exhibited focal HS within a single hemisphere (six right; four left). Focal HS was always found in the anterior hippocampus, between the pes hippocampus and the level of the lateral geniculate nucleus. Focal HS was commonly located at the junction of CA2 and CA1 (seven cases). Interestingly, there were no cases with focal HS in both cerebral hemispheres.

### TDP-43 immunostaining

TDP-43 immunostaining was carried out on both hemispheres of a subset of cases representing intact hippocampus (seven cases), IVD without HS (six cases), AD without HS (18 cases), and HS with and without comorbidities (14 cases). TDP-43-positive neurons of the dentate granule cell layer were found in 11/18 (61%) AD cases and 13/14 (92%) HS cases. No positive neurons were found in the intact or IVD cases. For the 14 cases of HS, the laterality of HS and the laterality of TDP-43-positive inclusions were largely congruent; 10/11 bilateral HS cases were positive bilaterally. Numbers of TDP-43 inclusions were highest in the bilateral HS cases (Table 1), where on average they exceeded 100 per hemisphere and were considerably higher than in the focal HS cases (mean = 10 per hemisphere) or AD cases (mean = 34 per hemisphere).

### MRI hippocampal volume

**Figure 3:**
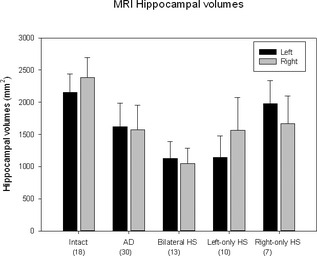
Bar chart illustrating left and right hippocampal volumes computed from MRI for intact hippocampi, AD without HS, bilateral HS, left-only HS, and right-only HS.

To explore whether MRI might be helpful in differentiating HS from AD in clinical settings, ANOVA was conducted separately for right and left HVs from MRIs closest to death (mean interval between MRI and death = 3.0 ± 2.1 years). Comparisons were made between the following groups: (1) cases with histologically intact hippocampi (*n* = 18), (2) AD without HS (*n* = 30), (3) bilateral HS without AD (*n* = 13), (4) left-sided HS (*n* = 10), and (5) right-sided HS (*n* = 7). Cases designated ‘Intact’ were those without significant neuropathology: HS = 0, Braak and Braak score ≤III, and CERAD ratings of none or sparse. Cases in the AD group were defined by Braak and Braak score ≥V, CERAD ratings of moderate or frequent, HS = 0, and DLB score = 0, and were without other pathological comorbidities. HS cases have Braak and Braak score ≤III, and CERAD ratings of none or sparse (*n* = 13) (Fig. 3).

Analysis of variance showed significant group differences in hippocampal MRI volumes (*P* < 0.001). Post hoc comparisons showed 32% greater atrophy in bilateral HS compared with AD (*P* < 0.001). There were also statistically significant differences between intact versus AD and intact versus HS for both right and left hippocampi (*P* < 0.001). On average, volumes for HS hippocampus were 52% and AD volumes were 30% less than intact hippocampi. As one might expect, for left-only HS, the left HV was smaller than the right (*P* = 0.002), and for right-only HS, the right volume was smaller than the left (*P* = 0.04).

As noted previously in the literature (Wang et al. 2003; Lye et al. 2004), in the cases with intact hippocampi, the right hippocampus was significantly larger than the left hippocampus (*P* = 0.009). The right HV for right-only HS was 31.3% larger than the left HV for left-only HS (*P* = 0.01), which may reflect underlying hemispheric asymmetries found in normal controls. Of interest, in right- or left-only HS cases, the MRI volumes in the contralateral and apparently uninvolved hippocampus were smaller than normal. For left-only HS cases, the right HV was significantly less than for normal controls (*P* = 0.0002). For right-only HS cases, there was a trend showing smaller volume of the left hippocampus compared with controls (*P* = 0.07). No statistically significant right versus left differences was found for either bilateral HS or AD.

## Discussion

HS may be a dramatic finding at autopsy that often escaped clinical recognition and diagnosis during a given subject's lifetime. In this convenience sample enriched for SIVD, AD, and normal aging, we found HS in 24.5% of autopsy cases. In our 31 cases, the laterality of HS showed significant variability: 45% were bilateral, 32% left-sided, and 23% right-sided. The frequency of HS in our study was nearly double the 12% previously reported in a community-based dementia autopsy series (Leverenz et al. 2002). Compared with the literature, our sample was intentionally enriched for SIVD, however, HS was found in roughly equal proportions in AD and SIVD (24% and 29%, respectively). More likely, the higher frequency of HS in our sample reflects the systematic examination of both hippocampi throughout their rostral–caudal extent in the majority of our cases. Indeed, based on our findings, the prevalence of HS will be underestimated by at least 25% if only one cerebral hemisphere is examined.

We initially defined complete HS as involvement across multiple coronal sections. In fact, however, we found that whenever there was a significant loss of pyramidal neurons accompanied by severe gliosis throughout the CA1 and/or the subiculum in one section, it could be seen in all sections throughout the rostral–caudal extent of that hippocampus. By contrast, in cases which were found to be focal, HS was seen only in a portion of CA1 or subiculum and only at a single level. In 10 (32%) cases, HS was focal in one hemisphere only (there were no cases with bilateral focal HS). Focal HS was always found in the anterior hippocampus, between the pes hippocampus and the level of the lateral geniculate nucleus, and most commonly located at the junction of CA2 and CA1 (seven cases). In screening for HS if tissue samples are limited, priority should be given to examining the anterior hippocampus.

As reported by others (Dickson et al. 1994; Jellinger 1994; Leverenz et al. 2002), we found HS associated with a number of comorbidities including AD, VaD, FTLD, and DLBD. Community-based autopsy studies have shown that mixed neuropathologies are found in 50% of dementia cases and 20% of nondemented cases (Schneider et al. 2007). Thus, it is possible that the overlap among pathologies simply reflects the co-occurrence of common entities in late life. Although AD was the most common neuropathologic diagnosis in our series, the proportions of AD and IVD cases harboring concomitant HS were similar, supporting the hypothesized chance co-occurrence of common entities.

The pathogenesis of HS remains unknown, with both ischemic and neurodegenerative theories proposed. Previous studies have observed associations between HS and vascular risk factors. Leverenz et al. noted that HS cases were more likely than AD to have had a history of stroke (56% vs. 25%) or hypertension (56% vs. 40%), evidence of small vessel disease (25% vs. 6%), but less likely to have had diabetes mellitus (0% vs. 22%; Leverenz et al. 2002). We have also noted associations between HS and a history of hypertension (Towfighi et al. 2008).

Investigators have observed a high frequency of HS in some forms of FTLD (Blass et al. 2004; Hatanpaa et al. 2004; Lippa and Dickson 2004), leading to the hypothesis that HS is neurodegenerative in origin. For example, patients with familial mutations in the progranulin gene show 50% reductions in plasma granulin expression, high prevalence of HS, and abundant intracytoplasmic TDP-43 inclusions (Rademakers et al. 2008). We are not able to comment on HS in FTLD, as FTLD is an exclusion criterion in the IVD program project, but one of the two cases of FTLD which was discovered incidentally at autopsy showed TDP-43-positive inclusions (case 16, Table 1).

More recently, TDP-43 inclusions has been reported in up to half of AD cases (Arai et al. 2009; Bigio et al. 2010), and thus are no longer considered specific for FTLD. We found TDP-43 inclusions in 93% of HS cases, including pure HS, and HS with various types of other pathologies. We also found inclusions in AD but not in pure IVD or controls. One can only speculate on the relationship between the presence of TDP-43 inclusions in the dentate granule cells and the loss of neurons and accompanying gliosis of HS in the CA1 and subiculum. The pathogenetic role of TDP-43 inclusions in disease has not yet been established. However, the finding of TDP-43 inclusions in 93% of HS cases irrespective of concomitant pathology and the absence of TDP-43 inclusions in pure IVD would be consistent with an underlying neurodegenerative rather than vascular mechanism.

Not surprisingly, MRI HVs were the smallest for bilateral HS cases, where atrophy exceeded that observed in AD (Fig. 3; Zarow et al. 2011). Compared with controls, we observed a 52% volume loss in bilateral HS cases compared with 30% loss in AD cases (Fig. 3). In this study, we extend observations regarding HVs to cases with unilateral HS. Interestingly, in unilateral HS cases, the contralateral hippocampus also shows evidence of volume loss on MRI. Reduction in HV in contralateral hippocampus was statistically significant in the left-sided HS cases (*P* < 0.0002) and showed a similar trend in the right-sided HS cases (*P* < 0.07). We and others have observed that the right hippocampus is larger than the left in normal controls (Wang et al. 2003; Lye et al. 2004). Developmental differences between the right versus left hippocampus or small sample size may contribute to these subtle hemispheric asymmetries. Our findings in cases of unilateral HS suggest that MRI volume may be more sensitive than pathology to early changes of HS or may be detecting transynaptic structural changes due to reduced cross talk between the two hippocampi. Future evidence-based studies are warranted to determine whether severity of atrophy can be used to distinguish individual cases of HS from AD.
